# Turkey adenovirus 3: ORF1 gene sequence comparison between vaccine-like and field strains

**DOI:** 10.1007/s11259-023-10148-4

**Published:** 2023-06-08

**Authors:** Giulia Quaglia, Antonietta Di Francesco, Elena Catelli, Giulia Mescolini, Caterina Lupini

**Affiliations:** Department of Veterinary Medical Sciences, University of Bologna, Via Tolara di Sopra, 50, Ozzano dell’Emilia (BO), 40064 Italy

**Keywords:** Turkey adenovirus 3, Turkey haemorrhagic enteritis, Molecular analysis, Phylogenetic analysis

## Abstract

Haemorrhagic enteritis is an economically significant disease reported in the majority of the countries where turkeys are raised intensively; it is caused by Turkey adenovirus 3 (TAdV-3). The aim of this study was to analyse and compare the ORF1 gene 3′ region from turkey haemorrhagic enteritis virus (THEV) vaccine-like and field strains in order to develop a molecular diagnostic method to differentiate the strains from each other. Eighty samples were analysed by sequencing and phylogenetic analyses using a new set of polymerase chain reaction (PCR) primers targeting a genomic region spanning the partial ORF1, hyd and partial IVa2 gene sequences. A commercial live vaccine was also included in the analysis. The results showed that 56 of the 80 sequences obtained in this study showed ≥99.8% nucleotide identity with the homologous vaccine strain sequence. Three non-synonymous mutations – ntA1274G (aaI425V), ntA1420C (aaQ473H) and ntG1485A (aaR495Q) – were detected in the THEV field strains but not in the vaccine strain. Phylogenetic analysis confirmed the clustering of the field and vaccine-like strains in different phylogenetic branches. In conclusion, the method employed in this study could be a useful tool towards making a correct diagnosis. The data could contribute to the knowledge of field distribution of THEV strains and increase the limited existing information available on native isolates around the world.

**Supplementary information:**

The online version contains supplementary material available at 10.1007/s11259-023-10148-4.

## Introduction

Haemorrhagic enteritis (HE) is an economically significant disease reported in the majority of the countries where turkeys are raised intensively. It is caused by the virulent variants of turkey haemorrhagic enteritis virus (THEV) or Turkey adenovirus 3 (TAdV-3), a ubiquitous double-stranded DNA virus that belongs to the genus *Siadenovirus* of the family *Adenoviridae* (Marek et al. 2014). THEV strains are classified as virulent and avirulent according to the clinical features induced in the host. The virulent strains cause depression, gastrointestinal haemorrhage and splenomegaly in turkeys older than 4 weeks, with mortality rates of up to 80% in maternal antibody–free turkeys (Rautenschlein et al. 2020), while the avirulent strains produce no intestinal symptoms (Domermuth et al. 1977, 1979; Silim et al. 1978). Both virulent and avirulent variants infect B cells in the spleen and macrophages, inducing immunosuppression and potentially fatal secondary bacterial infections (Smyth and McNulty 2008). The extensive use of live vaccines prepared with naturally occurring avirulent THEV strains (Domermuth et al. 1977) or cell culture–adapted strains (Fadly et al. 1985), as well as natural infections with avirulent THEV field strains, has decreased the incidence of HE outbreaks. However, the economic losses resulting from secondary bacterial infections favoured by immunosuppression should not be underestimated (Palya et al. 2007; Giovanardi et al. 2014; Alkie et al. 2017; Rautenschlein et al. 2020; Palomino-Tapia et al. 2020). THEV has a linear, highly stable, double-stranded DNA genome of 26.3 kb. Computational analysis of the THEV genome indicates the presence of 23 genes/ORFs, 18 of which are homologous to the common family genes located at the expected genomic positions – that is, IVa2, DNA-dependent DNA polymerase (pol), pTP, 52 K, pIIIa, III (penton base), pVII, pX, pVI, II (hexon), endoprotease (EP), DNA-binding protein (DBP), 100 K, 33 K, pVIII, and IV (fiber), 22 K and U exon. The remaining five ORFs are putative and genus-specific – ORF1 (sialidase), hyd, E3, ORF7 and ORF8 (Davison et al. 2003; Beach et al. 2009b). Virulent and avirulent THEV variants show 99.9% nucleotide sequence homology. Sequencing ORF1, E3 and the fiber knob domain has been proposed to differentiate vaccine-like and field THEV strains, because sequence variations in these genes in THEV strains with different phenotypes suggest that they are the key factors affecting virulence (Beach et al. 2009b). Alternatively, whole genome sequencing may be a useful tool to differentiate between vaccine-like and field strains, but it requires a high viral DNA concentration, which is usually obtained by viral propagation (Palomino-Tapia et al. 2020). The aim of this study was to analyse and compare the ORF1 gene 3′ region from THEV vaccine-like and field strains in order to develop a molecular diagnostic method to differentiate these strains from each other. Furthermore, this study attempts to provide a comprehensive update on THEV epidemiological situation in Europe, based on the results of the molecular and phylogenetic analysis performed on samples collected from 2016 to 2022 in different countries.

## Materials and methods

### Sample collection and Polymerase Chain Reaction (PCR)

This study focused on a total of 80 samples submitted between 2016 and 2022 to the Department of Veterinary Medical Sciences of the University of Bologna for routine diagnosis and tested positive for THEV using a molecular analysis according to Hess et al. (1999). The samples, consisting of spleen fragments, cloacal swabs or imprints of these on FTA cards, were collected from naturally deceased birds. Lesions referable to THEV infection, such as spleen enlargement, were occasionally reported. The samples originated from different turkey farms located in Italy (*n* = 28,), France (*n* = 21), Spain (*n* = 7), the United Kingdom (*n* = 15), Croatia (*n* = 8) and Germany (*n* = 1). Information about the collection date, country, type of sample, bird age and possible vaccination were recorded when available (Table S1). The strains were named using the following nomenclature: THEV/country of origin (Italy = IT; France = FR; Spai*n* = ES; the United Kingdom = UK; Croatia = HR; Germany = DE)/host species (Turkey = TY)/sample ID number/year of collection. In addition, the commercial THEV live attenuated vaccine DINDORAL SPF (Boehringer Ingelheim Animal Health, Lyon, France), used in Europe and containing the Domermuth strain, was included in the analysis. DNA was extracted from samples using the commercial NucleoSpin® Tissue kit (MACHEREY-NAGEL GmbH & Co. KG, Düren, Germany) according to the manufacturer’s instructions. All samples were analysed by PCR, using a new set of primers (ORF1-3 F: 5′-CAGGGTAGCGCTTTGTCACT-3′ and ORF1-3R: 5′-GAAAGAAAAACAAAAACGCATGT-3′). These primers target the 3′ region of the ORF1 gene, the entire hyd gene and a partial portion of 5′ region of the IVa2 gene, amplifying the genome at nucleotides (nt) 1512–2369 to produce a final product of 857 base pairs (bp). The amplification was performed by adding 3 µL of DNA to a 22-µL reaction mixture containing 0.125 µL of GoTaq G2 Flexi DNA Polymerase (Promega, Madison, WI, USA), 5 µL of 5X Green GoTaq Flexi Buffer, 1.75 µL of MgCl2 solution, 0.5 µL of dNTPs, 13.625 µL of H2O for molecular biology, 0.5 µL of forward primer (10 µM) and 0.5 µL of reverse primer (10 µM). The PCR thermocycler conditions included initial denaturation (95 °C for 2 min) followed by 40 amplification cycles at 95 °C for 40 s, 52 °C for 40 s and extension at 72 °C for 1 min. A final extension at 72 °C for 5 min completed the reaction. The PCR products were separated on agarose gel (1.5%) at 100 V and 400 mA for 40 min, stained with MIDORIgreen Advance (NIPPON Genetics Europe, Düren, Germany) and then visualised under ultraviolet light.

### Sequence and phylogenetic analysis

The obtained amplicons were purified using the ExoSAP-IT™ Express PCR Product Cleanup Kit (Thermo Fisher Scientific, Waltham, MA, USA) following the manufacturer’s instructions, and sequenced in both directions by Macrogen Europe (Amsterdam, the Netherlands). The nucleotide sequences were assembled and edited using the BioEdit Sequence Alignment Editor, Version 7.2.5.0 (Tom Hall, Ibis Therapeutics, Carlsbad, CA, USA), then aligned and compared using Clustal W software (Thompson et al. 1994) to publicly available homologous whole or partial genome sequences of THEV strains (Table S2) obtained using the BLAST server in GenBank (Sayers et al. 2020).

The phylogenetic trees based on nucleotide and amino acid sequences were constructed with the Maximum Likelihood method using MEGA X (Kumar et al. 2018). Nodes of the tree with bootstrap values obtained with 1000 replicates ≥ 70 were considered significant.

### Accession numbers

Sequences obtained in this study were submitted to the GenBank database are available under the following accession numbers: OQ412883–OQ412963.

## Results

**Fig. 1:**
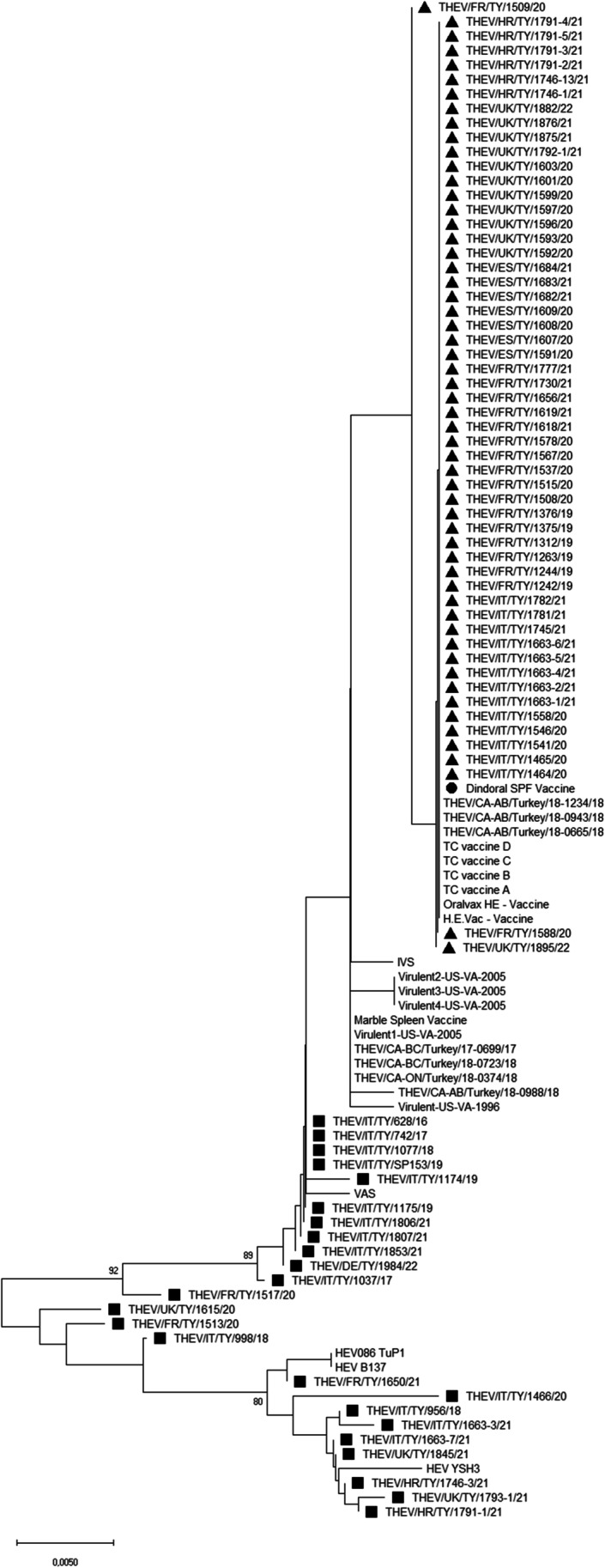
Phylogenetic tree based on the turkey haemorrhagic enteritis virus (THEV) nucleotide sequences obtained in this study and THEV references retrieved from GenBank. The THEV strains obtained in this study and considered the vaccine-like strains are marked with a black triangle. The THEV strains obtained in this study and considered the field strains are marked with a black square. The DINDORAL SPF vaccine is marked with a black circle. Only bootstrap values ≥ 70 are reported. The tree is drawn to scale, with branch lengths measured in the number of substitutions per site

**Fig. 2:**
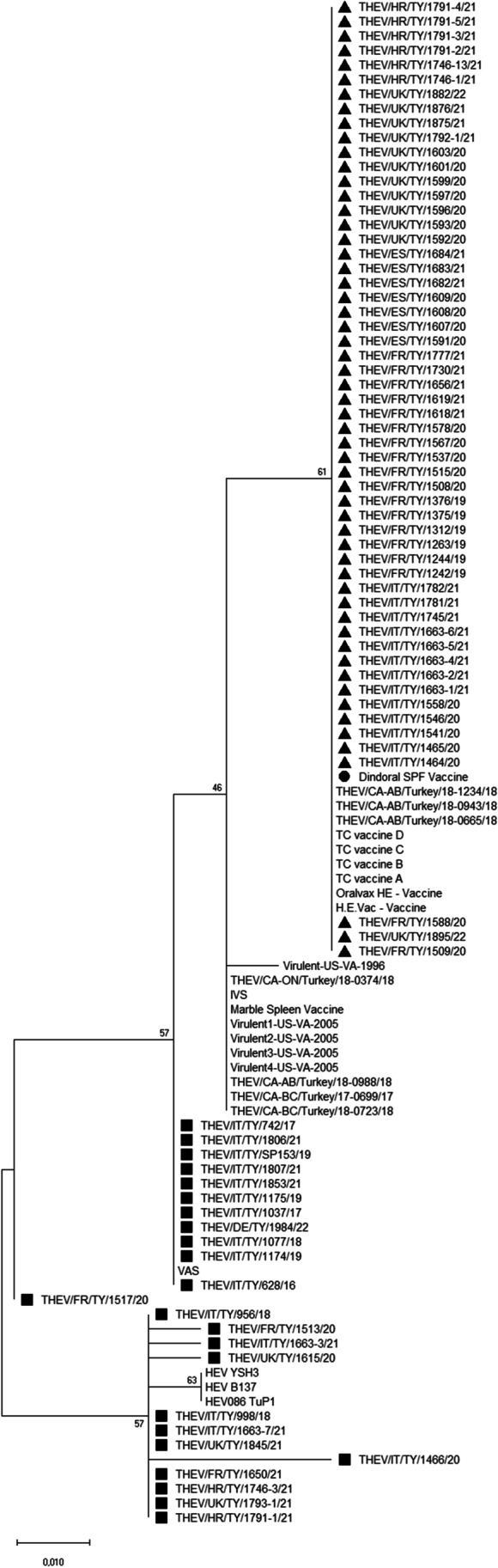
Phylogenetic tree based on the partial amino acid sequences of the ORF1 gene of the turkey haemorrhagic enteritis virus (THEV) strains analysed in this study and the reference strains retrieved from GenBank. The THEV strains obtained in this study and considered the vaccine-like strains are marked with a black triangle. The THEV strains obtained in this study and considered the field strains are marked with a black square. The DINDORAL SPF vaccine is marked with a black circle. The tree is drawn to scale, with branch lengths measured in the number of substitutions per site

All 80 examined samples produced an amplicon of the expected size. The sequences obtained included a fragment of the 3′ region of the ORF1 gene (441 nt), the complete hyd gene (227 nt) and a fragment of the 5′ region of the IVa2 gene (35 nt). The sequences showed 96.5–100% nucleotide identity when compared to each other and 95.6–100% nucleotide identity when compared with the nucleotide sequence of DINDORAL SPF vaccine. In particular, 56 of the 80 new sequences showed ≥ 99.8% nucleotide identity with the homologous vaccine strain sequence. The comparison of the sequences obtained in the present study with those available for THEV strains and the DINDORAL SPF vaccine showed the presence of 22 unique or shared mutations in the analysed ORF1 region (from nt 1512 to nt 1953), of which 9 were synonymous and 13 were non-synonymous (Table S3). In particular, eight Italian THEV variants (THEV/IT/TY/628/16, THEV/IT/TY/742/17, THEV/IT/TY/1037/17, THEV/IT/TY/998/18, THEV/IT/TY/1077/18, THEV/IT/TY/1174/19, THEV/IT/TY/1175/19 and THEV/IT/TY/1853/21) shared between them three non-synonymous mutations in the ORF1 region: ntA1274G (amino acid (aa) I425V), ntA1420C (aaQ473H) and ntG1485A (aaR495Q). These variants were from Italian turkey flocks where live vaccination was not performed, neither at the time of sample collection nor in previous production cycles, and hence they could be defined as field strains. The three substitutions were shared with other sequences obtained in this study from Italy as well as from other countries (*n* = 6 from Italy, *n* = 3 from France, *n* = 3 from the United Kingdom, *n* = 2 from Croatia and *n* = 1 from Germany) and the publicly available THEV sequences attributed to field strains (Beach et al. 2009b; Alkie et al. 2017; Palomino-Tapia et al. 2020; Gerber et al. 2022). Regarding the hyd and partial IVa2 gene sequences obtained, only spotted substitutions were observed in some strains when compared with the vaccine. In particular, in the nucleotide sequences of the hyd gene, there were six-point mutations, one of which not synonymous (ntT220G, aaF74V). In the partial IVa2 gene analysed, there was one non-synonymous point mutation (ntT7A, aaN11Y) in four different variants. The phylogenetic trees based on the nucleotide and amino acid sequences obtained in this study and those of reference strains are shown in Figs. 1 and 2, respectively. The THEV variants are grouped into two main clusters: 56 strains sequenced in the study clustered with the DINDORAL SPF vaccine and other THEV vaccines or vaccine-like strains retrieved from GenBank, while 24 strains clustered with strains retrieved worldwide and considered to be field strains. There was no geographical clustering in the phylogenetic trees.

## Discussion

The difficulty in differentiating between THEV vaccine and field strains has been acknowledged, considering the 99.9% nucleotide sequence homology between virulent and avirulent THEV strains (Beach et al. 2009b; Alkie et al. 2017). In the present study, 80 samples previously shown to be THEV positive during routine diagnosis were analysed by sequencing and phylogenetic analyses using a new set of PCR primers targeting a genomic region spanning the partial ORF1, full hyd and partial IVa2 gene sequences. The commercial live vaccine DINDORAL SPF was also included in the analysis. The analysis herein performed allowed the development of a molecular diagnostic method to differentiate field and vaccine-like strains and to contribute to increase the limited knowledge of the molecular characteristics of THEV strains detected worldwide.

Based on sequence analysis of the strains detected in this study compared with the DINDORAL SPF vaccine and other THEV reference sequences retrieved from GenBank, there were only single point mutations. There were no deletions or insertions in the amplified region of the genome. Interestingly, eight Italian THEV strains showed three-point mutations in the 3′ region of the ORF1 gene that were not present in DINDORAL SPF vaccine. The anamnestic data suggested to consider these particular strains definitely of field origin as they were from Italian turkey flocks in which live THEV vaccination was not performed, neither at the time of sample collection nor in previous production cycles. Of note, the live attenuated vaccine DINDORAL SPF (Domermuth strain) was introduced in Italy at the end of 2018 only in some Italian regions with a temporary import permit. The THEV Italian strains showing the three mutations were from turkey flocks located in Italian regions in which the use of the vaccine had not been authorised. These substitutions could be used as genetic markers allowing THEV variants to be characterised as field or vaccine like. Furthermore, these mutations were related to significant amino acid variations in the ORF1 gene, a genomic region previously thought to play a role in viral fitness, pathogenicity and possibly immune evasion (Beach et al. 2009b; Alkie et al. 2017). In particular, the field strains showed in the sialidase protein, coded by ORF1 gene, the presence of valine (amino acid position 425), histidine (amino acid position 473) and glutamine (amino acid position 495) instead of isoleucine, glutamine and arginine respectively, which are in the commercial vaccine and vaccine-like strains.

According to our study, the presence of these three mutations has been mentioned in previous studies in which the authors analysed sequences of strains sampled in different geographical areas (Alkie et al. 2017; Palomino-Tapia et al. 2020; Beach et al. 2009b; Gerber et al. 2022). Alkie at al. (2017) performed a molecular characterisation of THEV strains detected in vaccinated commercial turkey flocks in Germany from samples collected from 2008 to 2012. Sequence analysis showed the presence of the three above-mentioned amino acid variations between the commercial vaccine strains analysed and the four strains proposed to be of field origin and correlated with outbreaks of disease referable to THEV infection. Gerber et al. (2022) reported the same substitutions in field strains in a molecular characterisation survey of THEV collected from commercial turkey and meat chicken flocks in Australia. In the phylogenetic analysis, the Australian field strains formed a separate cluster compared with the THEV vaccine strains; similarly, the European field strains analysed in this study are grouped together in the same branch. Palomino-Tapia et al. (2020) reported a molecular characterisation of the whole genome of seven THEV strains obtained from clinical samples compared with the sequences of two vaccines strains available in Canada. The ORF1 region of the field strains had the same mutations observed in the present study (I425V, Q473H and R495Q). In addition, Beach et al. (2009b), in a broader analysis of genome segments obtained from cases of suspected clinical HE in United States, reported these single point mutations conserved in all analysed virulent strains. Furthermore, the vaccine strains analysed byPalomino-Tapia et al. (2020); Beach et al. (2009b) show 100% nucleotide identity in 3′ region of ORF1 gene with the European vaccine strain analysed in this study.

Phylogenetic analysis including reference strains from the aforementioned studies and strains analysed herein have confirmed the clustering of field and vaccine-like strains in different phylogenetic branches. The broader analysis of the strains collected in different European countries allowed us to characterise 56 vaccine-like strains and 24 field strains. All field strains possess the above-mentioned mutations in the ORF1 gene but otherwise only a nucleotide diversity of ≤ 0.3% relative to the DINDORAL SPF vaccine. The molecular characteristics of the field strains are shared between THEV sequences from different countries, suggesting that this molecular method may be useful in different geographical areas. So far, the only THEV sequences available from Europe were referred to Germany (Alkie et al. 2017); the present work contributes to broad the information related to the epidemiological status and the genetic characteristics of THEV variants circulating in Europe. Furthermore, phylogenetic analysis showed that the THEV field strains are not divided into clusters based on their geographic areas, confirming the molecular homogeneity of this virus. The low genetic variability of viruses belonging to the family *Adenoviridae* is well known (Pitcovski et al. 1998) and is confirmed by the molecular stability of the field strains analysed in the study, which had been collected in different countries and in a 6-year period.

The clinical and pathological histories of the farms in which the samples were collected reported only in a few cases the presence of splenomegaly and lesions referred to secondary bacterial infections. In these cases, the presence of THEV field strains was mainly reported, but the circulation of vaccine-like strains was also observed. Given that spleen enlargement is often observed in the animals after live THEV vaccination, additional studies are necessary to understand whether this lesion could be associated with immunosuppression, as previously suspected for certain vaccines strains (Palya et al. 2007; Alkie et al. 2017; Palomino-Tapia et al. 2020).

It is well known that the THEV vaccine strain can persist in turkeys for a long time in experimental conditions, up to 15 weeks after vaccination (Beach et al. 2009a). The present study increases the deepen understand of the vaccine kinetics; in particular, in turkey breeder farms the vaccine strain was detected in birds up to 60 weeks of age, suggesting the persistence of the applied vaccine in the field for a longer period than previously demonstrated. Adenoviral infection is associated with non-lytic persistence, and it has been also demonstrated in humans and animals species (Andiman and Miller 1982; Chu et al. 1992; Decaro et al. 2008). Latent viruses can remain inactive for long periods and then reactivate, resulting in viral shedding and constant production of antibodies, not associated with clinical disease (Beach et al. 2009a).

The circulation of THEV field strains in live vaccinated flocks has been observed in this study and has been reported by Alkie et al. (2017) and Palomino-Tapia et al. (2020). It is important to underline that the lack of vaccine efficacy in the field is frequently associated with improper handling of vaccine or the blanketing effect of a high level of maternally derived antibodies (Fadly and Nazerian 1989; Weier 2013; Düngelhoef et al. 2016). Furthermore, it is possible that in the same farm, field and vaccine THEV strains can circulate in different flocks, possibly due to multiple breaches in the applied biosecurity measures.

## Conclusion

In conclusion, the molecular characterisation of THEV strains and the differentiation between vaccine-like and field strains, by analysing the sequence of the 3′ region of the ORF1 gene, could be a useful tool towards a correct diagnosis and should be routinely applied, in order to better address the control strategies. Our data could contribute to the knowledge of the field distribution of THEV strains and increase the limited existing information available on native isolates around the world. In addition, full genome sequencing of field and vaccine strains may be necessary to identify mutations in other THEV genes, which may also contribute to characterise the circulating strains. An in vivo study could define the pathogenicity and the plausible role of the predicted amino acid residues on the virulence of the virus.

### Supplementary information

Below is the link to the electronic supplementary material.

Table S1 (PDF 170 KB)

Table S2 (PDF 128 KB)

Table S3 (PDF 121 KB)

## Data Availability

All data used and/or analysed during this study are included in this article and are available from the corresponding author on reasonable request. The sequences generated in this study are available in GenBank under accession numbers OQ412883–OQ412963.
