# Supplementary material for: Turkey adenovirus 3: ORF1 gene sequence comparison between vaccine-like and field strains
Source: Vet Res Commun. 2023 Jun 8;47(4):2307–13. doi: 10.1007/s11259-023-10148-4 (PMC10698090; doi:10.1007/s11259-023-10148-4)
Supplement: Supplementary file 1 — (PDF 170 KB) [file 11259_2023_10148_MOESM1_ESM.pdf]

TURKEY ADENOVIRUS 3: ORF1 GENE SEQUENCE COMPARISON BETWEEN VACCINE-LIKE AND FIELD STRAINS

Veterinary Research Communications

Giulia Quaglia[a], Antonietta Di Francesco[a], Elena Catelli[a], Giulia Mescolini[a] and Caterina Lupini[a]

[a] Department of Veterinary Medical Sciences, University of Bologna, Via Tolara di Sopra, 50, 40064, Ozzano dell’Emilia (BO), Italy

Correspondig author:

Giulia Quaglia, Department of Veterinary Medical Sciences, University of Bologna, Via Tolara di Sopra, 50, 40064, Ozzano dell’Emilia (BO), Italy; email: giulia.quaglia2@unibo.it

Table S1. Details of THEV samples analysed in this study.

ID Sample	Year of collection	Country	Type of sample/ Age of animal	Vaccination	NT Identity compared with DINDORAL-SPF (%)	Characterization	GenBank Accession no.
THEV/IT/TY/628/16	2016	Italy	Cloacal swabs	Not live vaccinated	99.5	Field strain	OQ412884
THEV/IT/TY/742/17	2017	Italy	Cloacal swabs 67 days of age	Not live vaccinated	99.6	Field strain	OQ412885
THEV/IT/TY/1037/17	2017	Italy	Spleens	Not live vaccinated	98.9	Field strain	OQ412886
THEV/IT/TY/956/18	2018	Italy	Cloacal swabs	Live vaccinated at 28 days of age	96.7	Field strain	OQ412887
THEV/IT/TY/998/18	2018	Italy	Cloacal swabs	Not live vaccinated	97.7	Field strain	OQ412888
THEV/IT/TY/1077/18	2018	Italy	Spleens	Not live vaccinated	99.7	Field strain	OQ412889
THEV/IT/TY/SP153/19	2019	Italy	Spleens	Live vaccinated	99.7	Field strain	OQ412890
THEV/IT/TY/1174/19	2019	Italy	Spleens	Not live vaccinated	99.5	Field strain	OQ412891
THEV/IT/TY/1175/19	2019	Italy	Spleens	Not live vaccinated	99.7	Field strain	OQ412892
THEV/IT/TY/1464/20	2020	Italy	Cloacal swabs	Live vaccinated	99.8	Vaccine-like strains	OQ412893
THEV/IT/TY/1465/20	2020	Italy	Cloacal swabs	Live vaccinated	100	Vaccine-like strains	OQ412894
THEV/IT/TY/1466/20	2020	Italy	Cloacal swabs	Live vaccinated at 26 days of age	96.7	Field strain	OQ412895
THEV/IT/TY/1541/20	2020	Italy	Spleens 138 days of age	Live vaccinated	99.8	Vaccine-like strain	OQ412896
THEV/IT/TY/1546/20	2020	Italy	Spleens 56 days of age	Live vaccinated at 22 days of age	100	Vaccine-like strain	OQ412897
THEV/IT/TY/1558/20	2020	Italy	Spleens 90 days of age	Live vaccinated at 35 days of age	100	Vaccine-like strain	OQ412898
THEV/IT/TY/1663-1/21					100	Vaccine-like strain	OQ412899
THEV/IT/TY/1663-2/21					100	Vaccine-like strain	OQ412900
THEV/IT/TY/1663-3/21					97.0	Field strain	OQ412901
THEV/IT/TY/1663-4/21	2021	Ital	Spleens 45 days of age	Live vaccinated	100	Vaccine-like strain	OQ412902
THEV/IT/TY/1663-5/21					100	Vaccine-like strain	OQ412903
THEV/IT/TY/1663-6/21					100	Vaccine-like strain	OQ412904
THEV/IT/TY/1663-7/21					97.0	Field strain	OQ412905
THEV/IT/TY/1745/21	2021	Italy	Spleens 84 days of age	Live vaccinated	100	Vaccine-like strain	OQ412906
THEV/IT/TY/1781/21	2021	Italy	Spleens	Live vaccinated	100	Vaccine-like strain	OQ412907
THEV/IT/TY/1782/21	2021	Italy	Spleens	Live vaccinated – 25 days of age	100	Vaccine like strain	OQ412908
THEV/IT/TY/1806/21	2021	Italy	Spleens 84 days of age	Live vaccinated	99.6	Field strain	OQ412909
THEV/IT/TY/1807/21	2021	Italy	Spleens 90 days of age	Live vaccinated	99.4	Field strain	OQ412910
THEV/IT/TY/1853/21	2021	Italy	Spleens 81 days of age	Not live vaccinated	99.4	Field strain	OQ412911
THEV/FR/TY/1242/19	2019	France	FTA cards spleen	Live vaccinated	100	Vaccine-like strain	OQ412912
THEV/FR/TY/1244/19	2019	France	FTA cards spleen	Live vaccinated	100	Vaccine-like strain	OQ412913
THEV/FR/TY/1263/19	2019	France	FTA cards spleen	Live vaccinated	100	Vaccine-like strain	OQ412914
THEV/FR/TY/1312/19	2019	France	FTA cards spleen	Live vaccinated	100	Vaccine-like strain	OQ412915
THEV/FR/TY/1375/19	2019	France	FTA cards spleen 42 days of age	Live vaccinated	99.8	Vaccine-like strain	OQ412916
THEV/FR/TY/1376/19	2019	France	FTA cards spleen 52 days of age	Live vaccinated	100	Vaccine-like strain	OQ412917
THEV/FR/TY/1508/20	2020	France	Spleens 54 days of age	Live vaccinated	100	Vaccine-like strain	OQ412918
THEV/FR/TY/1509/20	2020	France	Spleens 55 days of age	Live vaccinated	100	Vaccine-like strain	OQ412919
THEV/FR/TY/1513/20	2020	France	Spleens 84 days of age	Live vaccinated	97.1	Field strain	OQ412920
THEV/FR/TY/1515/20	2020	France	Spleens 84 days of age	Live vaccinated	100	Vaccine-like strain	OQ412921
THEV/FR/TY/1517/20	2020	France	Spleens 47 days of age	Live vaccinated	98.2	Field strain	OQ412922
THEV/FR/TY/1537/20	2020	France	Spleens 52 days of age	Live vaccinated	100	Vaccine-like strain	OQ412923
THEV/FR/TY/1567/20	2020	France	Spleens 84 days of age	Live vaccinated	99.8	Vaccine-like strain	OQ412924
THEV/FR/TY/1578/20	2020	France	FTA cards spleen 54 days of age	Live vaccinated	100	Vaccine-like strain	OQ412925
THEV/FR/TY/1588/20	2020	France	FTA cards spleen 77 days of age	Live vaccinated	100	Vaccine-like strain	OQ412926
THEV/FR/TY/1618/21	2021	France	Spleens 42 days of age	Live vaccinated	100	Vaccine-like strain	OQ412927
THEV/FR/TY/1619/21	2021	France	Spleens 43 days of age	Live vaccinated	100	Vaccine-like strain	OQ412928
THEV/FR/TY/1650/21	2021	France	FTA cards spleen 59 days of age	Live vaccinated	95.8	Field strain	OQ412929
THEV/FR/TY/1656/21	2021	France	Spleens 66 days of age	Live vaccinated	99.8	Vaccine-like strain	OQ412930
THEV/FR/TY/1730/21	2021	France	FTA cards spleen 77 days of age	Live vaccinated	100	Vaccine-like strain	OQ412931
THEV/FR/TY/1777/21	2021	France	FTA cards spleen 84 days of age	Live vaccinated	100	Vaccine-like strain	OQ412932
THEV/ES/TY/1591/20	2020	Spain	Cloacal swabs	Live vaccinated	100	Vaccine-like strain	OQ412933
THEV/ES/TY/1607/20	2020	Spain	Cloacal swabs 50 days of age	Live vaccinated	100	Vaccine-like strain	OQ412934

THEV/ES/TY/1608/20	2020	Spain	Cloacal swabs 50 days of age	Live vaccinated	100	Vaccine-like strain	OQ412935
THEV/ES/TY/1609/20	2020	Spain	Cloacal swabs	Live vaccinated	100	Vaccine-like strain	OQ412936
THEV/ES/TY/1682/21	2021	Spain	FTA cards spleen	Live vaccinated	100	Vaccine-like strain	OQ412937
THEV/ES/TY/1683/21	2021	Spain	FTA cards spleen 602 days of age	Live vaccinated	99.8	Vaccine-like strain	OQ412938
THEV/ES/TY/1684/21	2021	Spain	FTA cards spleen 280 days of age	Live vaccinated	99.8	Vaccine-like strain	OQ412939
THEV/UK/TY/1592/20	2020	United Kingdom	Cloacal swabs	Live vaccinated	100	Vaccine-like strain	OQ412940
THEV/UK/TY/1593/20	2020	United Kingdom	Cloacal swabs	Live vaccinated	100	Vaccine-like strain	OQ412941
THEV/UK/TY/1596/20	2020	United Kingdom	Cloacal swabs 62 days of age	Live vaccinated	100	Vaccine-like strain	OQ412942
THEV/UK/TY/1597/20	2020	United Kingdom	Spleens 85 days of age	Live vaccinated	100	Vaccine-like strain	OQ412943
THEV/UK/TY/1599/20	2020	United Kingdom	Spleens 86 days of age	Live vaccinated	100	Vaccine-like strain	OQ412944
THEV/UK/TY/1601/20	2020	United Kingdom	Spleens 79 days of age	Live vaccinated	100	Vaccine-like strain	OQ412945
THEV/UK/TY/1603/20	2020	United Kingdom	Spleens 78 days of age	Live vaccinated	100	Vaccine-like strain	OQ412946
THEV/UK/TY/1615/20	2020	United Kingdom	Cloacal swabs 67 days of age	Live vaccinated	97.5	Field strain	OQ412947
THEV/UK/TY/1792-1/21	2021	United Kingdom	FTA cards cloacal swabs 76 days of age	Live vaccinated	99.8	Vaccine-like strain	OQ412948
THEV/UK/TY/1793-1/21	2021	United Kingdom	FTA cards cloacal swabs 72 days of age	Live vaccinated	95.6	Field strain	OQ412949
THEV/UK/TY/1845/21	2021	United Kingdom	FTA cards cloacal swabs 36 days of age	Live vaccinated at 14 days of age	96.3	Field strain	OQ412950
THEV/UK/TY/1875/21	2021	United Kingdom	FTA cards cloacal swabs 62 days of age	Live vaccinated at 14 days of age	100	Vaccine-like strain	OQ412951
THEV/UK/TY/1876/21	2021	United Kingdom	FTA cards cloacal swabs 63 days of age	Live vaccinated at 14 days of age	100	Vaccine-like strain	OQ412952
THEV/UK/TY/1882/21	2021	United Kingdom	FTA cards cloacal swabs 68 days of age	Live vaccinated at 14 days of age	100	Vaccine-like strain	OQ412953
THEV/UK/TY/1895/21	2021	United Kingdom	FTA cards cloacal swabs 58 days of age	Live vaccinated at 21 days of age	100	Vaccine-like strain	OQ412954
THEV/HR/TY/1746-1/21	2021	Croatia	Spleens 90 days of age	Live vaccinated at 28 days of age	100	Vaccine-like strain	OQ412955
THEV/HR/TY/1746-3/21					96.3	Field strain	OQ412956
THEV/HR/TY/1746-13/21					100	Vaccine-like strain	OQ412957
THEV/HR/TY/1791-1/21	2021	Croatia	Spleens 133 days of age	Live vaccinated	96.3	Field strain	OQ412958
THEV/HR/TY/1791-2/21					99.8	Vaccine-like strain	OQ412959
THEV/HR/TY/1791-3/21					99.8	Vaccine-like strain	OQ412960
THEV/HR/TY/1791-4/21					99.8	Vaccine-like strain	OQ412961
THEV/HR/TY/1791-5/21					100	Vaccine-like strain	OQ412962
THEV/DE/TY/1984/22	2022	Germany	Cloacal swabs 63 days of age	Live vaccinated	99.6	Field strain	OQ412963
