# Supplementary material for: Turkey adenovirus 3: ORF1 gene sequence comparison between vaccine-like and field strains
Source: Vet Res Commun. 2023 Jun 8;47(4):2307–13. doi: 10.1007/s11259-023-10148-4 (PMC10698090; doi:10.1007/s11259-023-10148-4)
Supplement: Supplementary file 2 — (PDF 128 KB) [file 11259_2023_10148_MOESM2_ESM.pdf]

TURKEY ADENOVIRUS 3: ORF1 GENE SEQUENCE COMPARISON BETWEEN VACCINE-LIKE AND FIELD STRAINS

Veterinary Research Communications

Giulia Quaglia[a], Antonietta Di Francesco[a], Elena Catelli[a], Giulia Mescolini[a] and Caterina Lupini[a]

[a] Department of Veterinary Medical Sciences, University of Bologna, Via Tolara di Sopra, 50, 40064, Ozzano dell'Emilia (BO), Italy

Correspondig author:

Giulia Quaglia, Department of Veterinary Medical Sciences, University of Bologna, Via Tolara di Sopra, 50, 40064, Ozzano dell'Emilia (BO), Italy; email: giulia.quaglia2@unibo.it

Table S2. Public THEV sequences included in this study.

Strains	GenBank Accession No.	Reference
Israel Virulent Strain (IVS)	AF074946	(Pitcovski et al. 1998)
Virginia Avirulent Strain (VAS)	AY849321	(Beach et al. 2009)
Virulent-US-VA-1996	DQ868929	
Virulent1-US-VA-2005	DQ868931	
Virulent2-US-VA-2005	DQ868932	
Virulent3-US-VA-2005	DQ868933	
Virulent4-US-VA-2005	DQ868934	
Marble spleen vaccine	DQ868930	
TC vaccine A	DQ868935	
TC vaccine B	DQ868936	
TC vaccine C	DQ868937	
TC vaccine D	DQ868938	
HEV086 TuP1	MK493402	(Gerber et al. 2022)
HEV YSH3	MK493405	
HEV B137	MK493406	
H.E.Vac - Vaccine	MT603863	(Palomino-Tapia et al. 2020)
Oralvax HE - Vaccine	MT603864	
THEV/CA-AB/Turkey/18-0943/18	MT603866	
THEV/CA-AB/Turkey/18-0988/18	MT603867	
THEV/CA-AB/Turkey/18-1234/18	MT603868	
THEV/CA-BC/Turkey/17-0699/17	MT603869	
THEV/CA-BC/Turkey/18-0723/18	MT603870	
THEV/CA-ON/Turkey/18-0374/18	MT603871	

References Table S2:

Beach NM, Duncan RB, Larsen CT, et al (2009) Comparison of 12 turkey hemorrhagic enteritis virus isolates allows prediction of genetic factors affecting virulence. J Gen Virol 90:1978–1985. https://doi.org/10.1099/vir.0.010090-0

Gerber PF, Spatz S, Gray P, et al (2022) Circulation and Molecular Characterization of Hemorrhagic Enteritis Virus in Commercial Turkey and Meat Chicken Flocks in Australia. Avian Dis 66:53–59. https://doi.org/10.1637/21-00095

Palomino-Tapia V, Mitevski D, Inglis T, et al (2020) Molecular characterization of hemorrhagic enteritis virus (HEV) obtained from clinical samples in Western Canada 2017–2018. Viruses 12: https://doi.org/10.3390/v12090941

Pitcovski J, Mualem M, Rei-Koren Z, et al (1998) The Complete DNA Sequence and Genome Organization of the Avian Adenovirus, Hemorrhagic Enteritis Virus. Virology 249:307–315. https://doi.org/10.1006/VIRO.1998.9336
