# Supplementary material for: Turkey adenovirus 3: ORF1 gene sequence comparison between vaccine-like and field strains
Source: Vet Res Commun. 2023 Jun 8;47(4):2307–13. doi: 10.1007/s11259-023-10148-4 (PMC10698090; doi:10.1007/s11259-023-10148-4)
Supplement: Supplementary file 3 — (PDF 121 KB) [file 11259_2023_10148_MOESM3_ESM.pdf]

# TURKEY ADENOVIRUS 3: ORF1 GENE SEQUENCE COMPARISON BETWEEN VACCINE-LIKE AND FIELD STRAINS

**Veterinary Research Communications**

Giulia Quaglia[a], Antonietta Di Francesco[a], Elena Catelli[a], Giulia Mescolini[a] and Caterina Lupini[a]

[a] Department of Veterinary Medical Sciences, University of Bologna, Via Tolara di Sopra, 50, 40064, Ozzano dell'Emilia (BO), Italy

**Correspondig author:**

Giulia Quaglia, Department of Veterinary Medical Sciences, University of Bologna, Via Tolara di Sopra, 50, 40064, Ozzano dell'Emilia (BO), Italy; email: giulia.quaglia2@unibo.it

**Table S3**. Sequence differences in ORF1; hyd and IVa2 genes.

X##Y; X corresponds to DINDORAL-SPF vaccine (consensus sequence) nucleotide/amino acid; ## to gene position, and Y to the changing nucleotide/amino acid; - corresponds to the same amino acid of consensus sequence.

| Nt mutation | AA mutation | Gene | Sequences affected |
|---|---|---|---|
| T1241C | F414L | | THEV/IT/TY/1663-3/21 |
| T1244C | S415P | | HEV086 TuP1; HEV YSH3; HEV B137; THEV/IT/TY/1663-3/21; THEV/IT/TY/1663-7/21; THEV/FR/TY/1517/20; THEV/FR/TY/1650/21; THEV/UK/TY/1615/20; THEV/UK/TY/1793-1/21; THEV/UK/TY/1845/21; THEV/HR/TY/1746-3/21; THEV/HR/TY/1791-1/21 |
| C1257T | T419I | | HEV086 TuP1; HEV YSH3; HEV B137; THV/IT/TY/1466/20; THEV/IT/TY/1663-3/21; THEV/IT/TY/1663-7/21; THEV/FR/TY/1513/20; THEV/FR/TY/1517/20; THEV/FR/TY/1650/21; THEV/UK/TY/1793-1/21; THEV/UK/TY/1845/21; THEV/HR/TY/1746-3/21; THEV/HR/TY/1791-1/21 |
| A1267C | - | | HEV086 TuP1; HEV YSH3; HEV B137; THEV/IT/TY/1663-3/21; THEV/IT/TY/1663-7/21; THEV/FR/TY/1650/21; THEV/UK/TY/1793-1/21; THEV/UK/TY/1845/21; THEV/HR/TY/1746-3/21; THEV/HR/TY/1791-1/21 |
| A1274G | I425V | | IVS; VAS; HEV086 TuP1; HEV YSH3; HEV B137; Virulent-US-VA-1996; Marble Spleen Vaccine; Virulent1-US-VA-2005; Virulent2-US-VA-2005; Virulent3-US-VA-2005; Virulent4-US-VA-2005; THEV/CA-AB/Turkey/18-0988/18; THEV/CA-BC/Turkey/17-0699/17; THEV/CA-BC/Turkey/18-0723/18; THEV/CA-ON/Turkey/18-0374/18; THEV/IT/TY/628/16; THEV/IT/TY/742/17; THEV/IT/TY/1037/17; THEV/IT/TY/956/18; THEV/IT/TY/998/18; THEV/IT/TY/1077/18; THEV/IT/TY/SP153/19; THEV/IT/TY/1174/19; THEV/IT/TY/1175/19; THEV/IT/TY/1466/20; THEV/IT/TY/1663-3/21; THEV/IT/TY/1663-7/21; THEV/IT/TY/1806/21; THEV/IT/TY/1807/21; THEV/IT/TY/1853/21; THEV/FR/TY/1513/20; THEV/FR/TY/1517/20; THEV/FR/TY/1650/21; THEV/UK/TY/1615/20; THEV/UK/TY/1793-1/21; THEV/UK/TY/1845/21; THEV/HR/TY/1746-3/21; THEV/HR/TY/1791-1/21; THEV/DE/TY/1984/22 |
| A1313C | K438Q | ORF1 | HEV086 TuP1; HEV YSH3; HEV B137; THEV/IT/TY/956/18; THEV/IT/TY/998/18; THEV/IT/TY/1466/20; THEV/IT/TY/1663-3/21; THEV/IT/TY/1663-7/21; THEV/FR/TY/1513/20; THEV/FR/TY/1650/21; THEV/UK/TY/1615/20; THEV/UK/TY/1793-1/21; THEV/UK/TY/1845/21; THEV/HR/TY/1746-3/21; THEV/HR/TY/1791-1/21 |
| C1379T | - | | HEV086 TuP1; HEV YSH3; HEV B137; THEV/IT/TY/956/18; THEV/IT/TY/1663-3/21; THEV/IT/TY/1663-7/21; THEV/FR/TY/1513/20; THEV/FR/TY/1650/21; THEV/UK/TY/1793-1/21; THEV/UK/TY/1845/21; THEV/HR/TY/1746-3/21; THEV/HR/TY/1791-1/21 |
| C1379G | L460V | | THEV/IT/TY/1466/20 |
| G1398A | G466D | | HEV086 TuP1; HEV YSH3; HEV B137; THEV/IT/TY/956/18; THEV/IT/TY/998/18; THEV/IT/TY/1466/20; THEV/IT/TY/1663-3/21; THEV/IT/TY/1663-7/21; THEV/FR/TY/1513/20; THEV/FR/TY/1517/20; THEV/FR/TY/1650/21; THEV/UK/TY/1615/20; THEV/UK/TY/1793-1/21; THEV/UK/TY/1845/21; THEV/HR/TY/1746-3/21; THEV/HR/TY/1791-1/21 |
| A1420C | Q473H | | VAS; HEV086 TuP1; HEV YSH3; HEV B137; THEV/IT/TY/628/16; THEV/IT/TY/742/17; THEV/IT/TY/1037/17; THEV/IT/TY/956/18; THEV/IT/TY/998/18; THEV/IT/TY/1077/18; THEV/IT/TY/SP153/19; THEV/IT/TY/1174/19; THEV/IT/TY/1175/19; THEV/IT/TY/1466/20; THEV/IT/TY/1663-3/21; THEV/IT/TY/1663-7/21; THEV/IT/TY/1806/21; THEV/IT/TY/1807/21; THEV/IT/TY/1853/21; THEV/FR/TY/1513/20; THEV/FR/TY/1517/20; THEV/FR/TY/1650/21; THEV/UK/TY/1615/20; THEV/UK/TY/1793-1/21; THEV/UK/TY/1845/21; THEV/HR/TY/1746-3/21; THEV/HR/TY/1791-1/21; THEV/DE/TY/1984/22 |
| A1433G | I478V | | HEV086 TuP1; HEV YSH3; HEV B137; THEV/IT/TY/956/18; THEV/IT/TY/998/18; THEV/IT/TY/1466/20; THEV/IT/TY/1663-3/21; THEV/IT/TY/1663-7/21; THEV/FR/TY/1513/20; THEV/FR/TY/1650/21; THEV/UK/TY/1615/20; THEV/UK/TY/1793-1/21; THEV/UK/TY/1845/21; THEV/HR/TY/1746-3/21; THEV/HR/TY/1791-1/21; |
| T1441G | I480M | | THEV/IT/TY/1466/20 |
| A1450G | - | | THEV/IT/TY/1174/19 |

| | | | |
|---|---|---|---|
| A1469G | I490V | ORF1 | HEV086 TuP1; HEV YSH3; HEV B137; THEV/IT/TY/956/18; THEV/IT/TY/998/18; THEV/IT/TY/1466/20; THEV/IT/TY/1663-3/21; THEV/IT/TY/1663-7/21; THEV/FR/TY/1513/20; THEV/FR/TY/1650/21; THEV/UK/TY/1615/20; THEV/UK/TY/1793-1/21; THEV/UK/TY/1845/21; THEV/HR/TY/1746-3/21; THEV/HR/TY/1791-1/21; |
| G1485A | R495Q | | IVS; VAS; HEV086 TuP1; HEV YSH3; HEV B137; Virulent-US-VA-1996; Marble Spleen Vaccine; Virulent1-US-VA-2005; Virulent2-US-VA-2005; Virulent3-US-VA-2005; Virulent4-US-VA-2005; THEV/CA-AB/Turkey/18-0988/18; THEV/CA-BC/Turkey/17-0699/17; THEV/CA-BC/Turkey/18-0723/18; THEV/CA-ON/Turkey/18-0374/18; THEV/IT/TY/628/16; THEV/IT/TY/742/17; THEV/IT/TY/1037/17; THEV/IT/TY/956/18; THEV/IT/TY/998/18; THEV/IT/TY/1077/18; THEV/IT/TY/SP153/19; THEV/IT/TY/1174/19; THEV/IT/TY/1175/19; THEV/IT/TY/1466/20; THEV/IT/TY/1663-3/21; THEV/IT/TY/1663-7/21; THEV/IT/TY/1806/21; THEV/IT/TY/1807/21; THEV/IT/TY/1853/21; THEV/FR/TY/1513/20; THEV/FR/TY/1517/20; THEV/FR/TY/1650/21; THEV/UK/TY/1615/20; THEV/UK/TY/1793-1/21; THEV/UK/TY/1845/21; THEV/HR/TY/1746-3/21; THEV/HR/TY/1791-1/21; THEV/DE/TY/1984/22 |
| T1533C | L511S | | THEV/IT/TY/1466/20 |
| G1582T | - | | HEV086 TuP1; HEV YSH3; HEV B137; THEV/IT/TY/956/18; THEV/IT/TY/998/18; THEV/IT/TY/1466/20; THEV/IT/TY/1663-3/21; THEV/IT/TY/1663-7/21; THEV/FR/TY/1650/21; THEV/UK/TY/1793-1/21; THEV/UK/TY/1845/21; THEV/HR/TY/1746-3/21; THEV/HR/TY/1791-1/21 |
| C1586T | - | | HEV086 TuP1; HEV YSH3; HEV B137; THEV/IT/TY/956/18; THEV/IT/TY/1466/20; THEV/IT/TY/1663-3/21; THEV/IT/TY/1663-7/21; THEV/FR/TY/1513/20; THEV/FR/TY/1517/20; THEV/FR/TY/1650/21; THEV/UK/TY/1615/20; THEV/UK/TY/1793-1/21; THEV/UK/TY/1845/21; THEV/HR/TY/1746-3/21; THEV/HR/TY/1791-1/21 |
| A1603T | - | | HEV086 TuP1; HEV YSH3; HEV B137; THEV/IT/TY/956/18; THEV/IT/TY/998/18; THEV/IT/TY/1466/20; THEV/IT/TY/1663-3/21; THEV/IT/TY/1663-7/21; THEV/FR/TY/1513/20; THEV/FR/TY/1517/20; THEV/FR/TY/1650/21; THEV/UK/TY/1615/20; THEV/UK/TY/1793-1/21; THEV/UK/TY/1845/21; THEV/HR/TY/1746-3/21; THEV/HR/TY/1791-1/21 |
| T1604C | - | | HEV YSH3; THEV/IT/TY/956/18; THEV/IT/TY/998/18; THEV/IT/TY/1466/20; THEV/IT/TY/1663-3/21; THEV/IT/TY/1663-7/21; THEV/UK/TY/1793-1/21; THEV/UK/TY/1845/21; THEV/HR/TY/1746-3/21; THEV/HR/TY/1791-1/21 |
| A1606G | - | | HEV086 TuP1; HEV YSH3; HEV B137; THEV/IT/TY/956/18; THEV/IT/TY/998/18; THEV/IT/TY/1466/20; THEV/IT/TY/1663-3/21; THEV/IT/TY/1663-7/21; THEV/FR/TY/1513/20; THEV/FR/TY/1650/21; THEV/UK/TY/1615/20; THEV/UK/TY/1793-1/21; THEV/UK/TY/1845/21; THEV/HR/TY/1746-3/21; THEV/HR/TY/1791-1/21 |
| C1609T | - | | HEV086 TuP1; HEV YSH3; HEV B137; THEV/IT/TY/956/18; THEV/IT/TY/1466/20; THEV/IT/TY/1663-3/21; THEV/IT/TY/1663-7/21; THEV/FR/TY/1650/21; THEV/UK/TY/1615/20; THEV/UK/TY/1793-1/21; THEV/UK/TY/1845/21; THEV/HR/TY/1746-3/21; THEV/HR/TY/1791-1/21 |
| A63G | - | Hyd | HEV086 TuP1; HEV YSH3; HEV B137; THEV/IT/TY/956/18; THEV/IT/TY/998/18; THEV/IT/TY/1466/20; THEV/IT/TY/1663-3/21; THEV/IT/TY/1663-7/21; THEV/FR/TY/1513/20; THEV/FR/TY/1517/20; THEV/UK/TY/1615/20; THEV/UK/TY/1845/21; THEV/HR/TY/1746-3/21; THEV/HR/TY/1791-1/21 |
| G66A | - | | THEV/IT/TY/1807/21; THEV/IT/TY/1853/21; |
| A75G | - | | THEV/FR/TY/1375/19 |
| A111C | - | | THEV/IT/TY/1466/20; THEV/FR/TY/1656/21; THEV/ES/TY/1683/21; THEV/ES/TY/1684/21 |
| A180C | - | | HEV086 TuP1; HEV YSH3; HEV B137; THEV/IT/TY/956/18; THEV/IT/TY/1466/20; THEV/IT/TY/1663-7/21; THEV/FR/TY/1517/20 |
| T220G | F74V | | HEV086 TuP1; HEV YSH3; HEV B137; THEV/IT/TY/956/18; THEV/IT/TY/998/18 |
| T7A | N11Y * | IVa2 | THEV/IT/TY/628/16; THEV/IT/TY/SP153/19; THEV/IT/TY/1174/19; THEV/IT/TY/1175/19 |

* RC: reverse complement.
